# Variation in skin barrier function throughout smoltification in Atlantic salmon (*Salmo salar*)

**DOI:** 10.3389/fphys.2026.1856527

**Published:** 2026-06-19

**Authors:** Darragh Doyle, Ana Silva Gomes, Kristina Sundell, Sigurd Handeland, Henrik Sundh

**Affiliations:** 1 Department of Biological and Environmental Sciences, University of Gothenburg, Gothenburg, Sweden; 2 Department of Biological Sciences, University of Bergen, Bergen, Norway

**Keywords:** claudin, skin, smoltification, tight junctions, transepithelial resistance, Ussing chamber

## Abstract

**Introduction:**

Smoltification involves distinct morphological, physiological and behavioral changes in Atlantic salmon, yet skin barrier function has not been functionally assessed throughout this transition.

**Method:**

We measured electrophysiological parameters at three life stages — parr, smolt, and post-smolt — to assess changes in skin permeability and transport capacity.

**Results:**

Parr skin showed significantly lower transepithelial resistance (higher permeability) and more negative short-circuit current than smolts under identical Ringer`s conditions. Seawater exposure elevated transepithelial potential in post-smolt relative to smolt, while freshwater exposure elevated short-circuit current in smolt relative to parr. No significant differences in mRNA expression of selected tight junction genes were found between life stages. We detected no significant correlation between skin permeability and any of the selected genes.

**Discussion:**

The results suggests that other claudin isoforms or posttranscriptional mechanisms underlie the observed functional changes. From a husbandry perspective, elevated skin permeability following seawater exposure highlights the importance of prioritizing skin health during and after smoltification, as skin function may be compromised at this time.

## Highlights

- Skin permeability differed significantly throughout smoltification.
- Active ion transport in the skin changes significantly during smoltification.
- There were no significant differences in the mRNA expression of *occludin*, *zo*-1, *cldns* - *3b*, *3c*, *7*, or *10e* throughout smoltification.
- No significant correlation between claudin expression and within-group skin permeability was detected.

## Introduction

For anadromous fish, the parr-smolt transformation (smoltification) is a period of major physiological and behavioral change (Björnsson et al., 2011), which highlights the challenges presented for these fish in both freshwater (FW) and seawater (SW). From an osmoregulatory perspective, in FW, the fish must compensate for the passive inflow of water and loss of ions by actively taking up ions from the surrounding water and producing a large amount of hypotonic urine (Krogh, 1938). In SW, the passive flows are reversed, and the fish must work to prevent the osmotic loss of water to the hypertonic environment through low urine production. Simultaneously, the fish must drink the SW and extract fluid from it, as well as excrete excess ions (Grosell, 2006). The differences in these complex and distinct needs are reflected in the changes that occur during smoltification, all of which pre-adapt the fish for life in SW. These pre-adaptations are especially apparent in the epithelia of the main osmoregulatory organs i.e., the intestine, kidney and gill. In the gill, SW chloride cells proliferate and abundantly express NKA (alpha 1b) and NKCC1 to facilitate active NaCl secretion, while in the intestine, NKA (alpha 1c), NKCC2 and NCC proliferate to drive ion-coupled water uptake (Sundell and Sundh, 2012; Grosell, 2006). Despite the roles of the gill and intestine being quite well established in relation to smoltification, the response and function of the skin during this important transitional life stage remains less clear.

The skin is one of the primary barriers that separates the internal environment of the fish from its external environment. As such, it is vital for maintaining homeostasis (Ángeles Esteban, 2012; Rakers et al., 2010). Barrier function in the teleost skin is a summation of several different tissues and processes. While mucus secreted by goblet cells in the epidermis is a main barrier for pathogens and antigens, the epidermis and associated tight junction complex acts as the physical barrier to limit ion and water flux (Gauberg et al., 2017). This cohesive layer also acts to prevent antigen and pathogen adherence and uptake (Guttman and Finlay, 2009). The permeability is regulated by environmental salinity in rainbow trout, as skin exposed to SW is far more permeable than skin exposed to FW (Doyle et al., 2022). During smoltification, several morphological and biochemical changes have previously been reported to occur in the skin. These include a thickening of the epidermis and an increase in goblet cell count (Karlsen et al., 2018). Additionally, immune function in the skin can decrease in the period immediately following SW transfer, but enhance in the months following (Karlsen et al., 2018). Alterations in the mucus-associated antimicrobial compounds have also been demonstrated (Fagan et al., 2003). Knowledge and possible correlations of these events to potential skin permeability changes during smoltification is currently lacking.

Barrier function in the skin and other epithelia is highly regulated by the tight junctions (TJs) - the defining characteristic of these barriers. This complex is composed of transmembrane proteins that form the intercellular barrier between the epithelial cells and connect indirectly to the actin cytoskeleton (Zihni et al., 2016). This regulates the movement of solutes between tissue compartments. Functional TJs include claudins, occludins, and Zonula Occludens (ZO) proteins, the latter of which act as a scaffold, connecting the claudins and occludins to the actin cytoskeleton (Hartsock and Nelson, 2008). The TJs perform two mutually exclusive functions. The first is a ‘fence’ function, whereby the transmembrane occludins and claudins connected to the actin cytoskeleton create and maintain the separation between apical and basolateral membrane domains by forming a barrier that restricts the movement of membrane proteins and lipids within the plasma membrane (Cummins, 2012). The second is the ‘gate’ function, where the claudins are the proteins mainly responsible for selectively facilitating or hindering the paracellular diffusion of solutes by creating size- and charge-selective pores in the intercellular space (Krause et al., 2008). TJs are also involved in signal transduction, which allows them to respond rapidly to environmental stimuli (Takano et al., 2014). Teleosts possess genes encoding for a broad range of claudins across a range of tissues — for example, 56 claudins have been described in the genome of *Fugu rubripes* (Loh et al., 2004) — and the differential expression of genes encoding for certain claudin isoforms suggests that certain isoforms either contribute to increased or reduced permeability, acting as pore-forming or barrier-forming elements for specific ions. This great diversity of claudins is likely necessary to dynamically regulate epithelial function in response to an ever-changing set of abiotic conditions (Kolosov et al., 2013). In the gill, smoltification and changes in salinity regulate the expression of TJ proteins (Chasiotis et al., 2012). For example, in Atlantic salmon, SW acclimation results in increased branchial expression of cldn-10e transcripts, suggested to function as a Na^+^ channel (Tipsmark et al., 2008), while the same pattern has been observed for cldns-10c and -10e in tilapia gill (Tipsmark et al., 2016), and an increase in cldns-10d and -10e was observed in the mummichog gill following SW transfer (Marshall et al., 2018).Claudin isoforms have been identified in the skin of salmonids, with a particularly extensive characterization in rainbow trout (Gauberg et al., 2017) and more limited coverage in Atlantic salmon (Sveen et al., 2016, Sveen et al., 2023). Despite this, the role of smoltification in modulating TJ expression in Atlantic salmon (or any other salmonid) skin has not yet been explored. The lack of focus on skin function during smoltification is likely attributed to the view that skin is a relatively impermeable barrier that contributes little in terms of active transport. However, this has been challenged in recent years and several active transport components have been described e.g. V-ATPase, NHE, Rh proteins (Doyle et al., 2022; Glover et al., 2013; Zimmer et al., 2014). The aim of this study was to assess whether skin barrier function is regulated during smoltification and after SW transfer in Atlantic salmon. Furthermore, we sought to relate skin barrier function to the expression of selected claudin isoforms, thereby linking TJ protein expression to actual barrier function.

## Methods

### Experimental fish and holding conditions

The full experimental design for the study is outlined in Gomes et al. (2025), along with a schematic of the timeline (**Supplementary Figure S1**). In brief, Atlantic salmon parr were obtained from Lerøy Sjøtroll AS (Kjærelva, Fitjar, Norway) and transferred to duplicate tanks at the Department of Biological Sciences (University of Bergen, Bergen, Norway). After 1 month of quarantine, the fish (98.6 ± 17.2 g) were moved into duplicated experimental tanks (80 fish per tank). The experimental tanks were 80 cm high with a diameter of 104 cm and had a 20 cm cylinder-shaped separator positioned in the center. The water depth was 55 cm. The salmon parr were acclimated for four weeks at a swimming speed of 0.5 body lengths/second (BL/s). Flow rate was monitored using a Flow Watch FW450 (General, USA). The winter signal (5 weeks of 12:12 L:D) was followed by 6 weeks at constant light to induce smoltification. During this time, the flow rate was increased to 1.0 BL/s. During the FW phase, the water temperature was 12.5 ± 0.2 °C and oxygen levels were kept above 80%. Fish were then transferred to 26.8 ppt brackish water (temperature at 9.9 ± 0.2 °C, and oxygen levels above 90%) for a total of 5 weeks where the swimming speed was kept at 0.5 BL/s. Sampling was carried out at three experimental time points, which corresponded to three discrete life history phases: the parr stage (at the end of the winter signal), the smolt stage (after 6 weeks of constant light), and the post-smolt stage (after 5 weeks in SW).

### Experimental protocol

For sampling, the fish were euthanized with an overdose (200 mg/L) of NaHCO_3_-buffered tricaine methanesulfonate (MS-222™; MSD Animal Health, Netherlands), after which length and weight were measured. Two skin sections were then carefully excised from the left dorsal flank of each fish and transferred to chilled Ringer`s solution to be used for Ussing chamber analysis. For parr and smolt in FW, the Ringer`s solution was made according to Sundell et al. (2003) modified for air and consisted of: mmol L−1: NaCl 140, KCl 2.5, CaCl_2_ 1.5, MgSO_4_*7H_2_O 0.8, HEPES 5, D-Glucose 10, L-Glutamine 20. For the post-smolt in brackish water, the Ringer`s solution was: mmol L−1: NaCl 150, KCl 2.5, CaCl_2_ 2.5, MgCl_2_*6H_2_O 1, HEPES 5, D-Glucose 10, L-Glutamine 20. The pH was set to 7.8 using 1.5 M Trizma base. During the excision, care was taken to ensure that no muscle tissue was attached to the skin. At this time, an additional small section of skin was excised and placed in RNAlater for use in gene expression analysis. The skin samples in RNAlater were stored at 4 °C overnight, before transfer to -20 °C for long term storage.

### Ussing chamber method

Skin barrier function was assessed using the Ussing chamber technique (UCC-401; UCC-Laboratories Ltd.) described by Sundell et al. (2003), and using modifications described by Sundell and Sundh (2012). In brief, this method measured several electrophysiological parameters from the excised skin tissue, which was mounted into and separating two half chambers (through a 0.75 cm^2^ aperture) containing identical or different salt solutions representing the external environment (apical side) or the internal environment (basal side). An air pump provided circulation, oxygenation and pH regulation to ensure viability of the excised skin for the duration of the experiment. The temperature of the chambers was kept at the same temperature as the experimental tanks using a water-cooled mantle.

The Ussing chamber experiments were conducted in two distinct phases. In the first phase, 4 mL of chilled Ringer’s solution was added to both the apical and basal chamber halves, creating isosmotic conditions with no concentration gradient across the tissue. Electrophysiological measurements were recorded every 5 minutes throughout a 30-minute acclimation period under these conditions. This phase allowed for direct comparison of skin barrier function across life stages (parr, smolt, and post-smolt) in the absence of any osmotic driving force.

In the second phase, to simulate *in vivo*-like conditions, the apical Ringer’s solution was removed and immediately replaced with either FW or brackish water, depending on the life stage being examined. For parr, the apical solution was replaced with FW. For smolt, the apical solution was replaced with either FW or brackish water. For post-smolt, the apical solution was replaced with brackish water. Electrophysiological measurements continued to be recorded every 5 minutes following this exchange. Data from the identical Ringer’s phase and the *in vivo*-like phase were treated as separate experimental conditions and were not directly compared to one another.

Alternating adaptive DC voltages (U) were applied to the mounted skin every 5 min using nitinol electrodes. The applied voltages generated corresponding currents (I) alternating between positive and negative currents of max 30 μA, to avoid electrical charging of the epithelium. The U/I pairs were fitted to a straight line using the least-square method. The slope of the line represented the transepithelial electrical resistance (TER). A pair of KCl electrodes in a 3 M KCl solution continuously measured the transepithelial potential difference (TEP) across the epithelium via a series of agar bridges. The short-circuit current (SCC) was calculated as SCC = -TEP/TER. The TER mainly reflects the integrity of the tissue, taking both transcellular and paracellular pathways into account, while the TEP reflects the net ion distribution across the epithelium as a result of the paracellular and transcellular transfer of ions. Finally, SCC is a measure of the net ion transport across the epithelium.

### RNA extraction and cDNA synthesis

Total RNA was isolated utilizing the RNeasy mini kit from Qiagen (Qiagen NV, Hilden, Germany). The skin samples stored in RNAlater were thawed at 4 °C overnight before extraction. For each skin section, the epidermis and scales were scraped away from the underlying dermis using glass slides. The epidermis and scales were placed in 600 µl of RLT plus buffer, lysed with the Tissuelyser II homogenizer (Qiagen NV, Hilden, Germany), and used for subsequent RNA extraction following the manufacturer’s instructions. RNA concentration was determined using the Nanodrop One/Onec spectrophotometer (Thermo Fisher Scientific, Waltham, MA, USA). The samples were then diluted with RNase-free water to achieve a uniform RNA concentration (500 ng/sample). Total RNA was subsequently used for cDNA synthesis with the iScript cDNA synthesis kit (Bio-Rad Laboratories, Richmond, CA, USA) according to the manufacturer’s protocol. The quality of cDNA was assessed using the Agilent TapeStation system (Agilent Technologies, Santa Clara, CA, USA).

### qPCR

In the current study, we selected the claudin isoforms that were shown to be regulated during smoltification and SW transfer in Atlantic salmon gills (Tipsmark et al., 2009; Tipsmark and Madsen, 2012). The specific gene primer pair (**Table 1**) efficiency was confirmed via a serial dilution (seven-point, 3-fold) of pooled cDNA from each sample. Based on the efficiency and R^2^, *cldns -3a* and *27a* were excluded from further analyses. qPCR was conducted using a 1:15 dilution, with the chosen dilution derived from the serial dilutions in the primer efficiency curve. SYBR Green served as the fluorescent intercalating agent. The qPCR was run for 40 cycles, each consisting of 10 seconds of denaturation at 95 °C and 30 seconds of annealing and extension at 60 °C. A melt curve was analyzed by gradually increasing the temperature from 60 °C to 95 °C over 20 minutes to confirm single-specific products. All reactions were performed on a CFX Connect Real-time PCR Detection System (Bio-Rad Laboratories, Hercules, CA, USA) and data were processed using Bio-Rad CFX Manager software (V 2.3). Gene expression values are presented as relative expression using the reference gene *ef-1a*, and calculated using the method described by Pfaffl (2001).

**Table 1:** Primer pair information for the genes used in the present study.

Gene	Name	Amplicon size (bp)	Efficiency (%)	R²	Primer sequence	Genbank accession number
*cldn-3a*	Claudin 3a	113	106.83	0.638	F: AGGGTTGGAGTTAGTGGGGA	XM014162769
					R: TGACGATGTTGCTGCCGATA	
*cldn-3b*	Claudin 3b	110	101.03	0.979	F: ATCCTGTGCTGTAGTTGCCC	XM014162770
					R: CTTTTGTCATAGCCGCTGGG	
*cldn-3c*	Claudin 3c	117	96.43	0.996	F: TCGGAGCCAAGTGTACCAAC	BK006383
					R: CCAAGGAAACGGGGATGAGG	
*cldn-7*	Claudin 7	91	96.17	0.996	F: GCCTTCCAGTGTGAGACCTAC	XM014195723
					R: AAAAGACCACGGAGACCACC	
*cldn- 10e*	Claudin 10e	95	108.76	0.967	F: ATCAAGGTGGCCTGGTACTG	BK006391
					R: GACCAGAGCACAGGGAAGTC	
*cldn-27a*	Claudin 27a	99	120.71	0.920	F: GACAGGTATCGTCGGCATCT	BK006400
					R: CCAGCCACAATACAGGCTCT	
*occludin*	Occludin	101	113.40	0.986	F: GACAGTGAGTTCCCCACCAT	XM014137436
					R: ATCTCTCCCTGCAGGTCCTT	
*zo-1*	Zonula occludens-1	119	113.57	0.983	F: CAAAGCCAGTGTATGCCAG	XM014175464.2
					R: CAGCTTCATACTCGGCCTGA	
*ef-1a*	Elongation factor 1 alpha	71	104.70	0.999	F: AGAACCATTGAGAAGTTCGAGAAG	AF321836
					R: GCACCCAGGCATACTTGAAAG	

### Statistical analyses

To assess differences in skin function between life stages, TER, TEP, and SCC were first compared under identical Ringer`s conditions using Welch’s ANOVAs and *post-hoc* Dunnett’s T3 tests for multiple comparisons. This allowed for direct comparison of parr, smolt and post-smolt. Welch’s tests were used to compare the effect of salinity on skin function at different life stages. Due to the small sample size, samples from the duplicate tanks were pooled for all statistical tests. Only groups exposed to common salinity were compared i.e. parr and smolt exposed to FW were compared and smolt and post-smolt exposed to SW were compared. For all pairwise comparisons, Welch tests were used. To assess the relationship between skin permeability and the mRNA expression of TJ proteins, Spearman rank correlation tests were used. Alpha was set at 0.05 for all tests. Normality was assessed using QQ plots. All analyses were carried out in GraphPad version 10.

## Results

### Electrophysiology under identical Ringer's conditions

**Figure 1:**
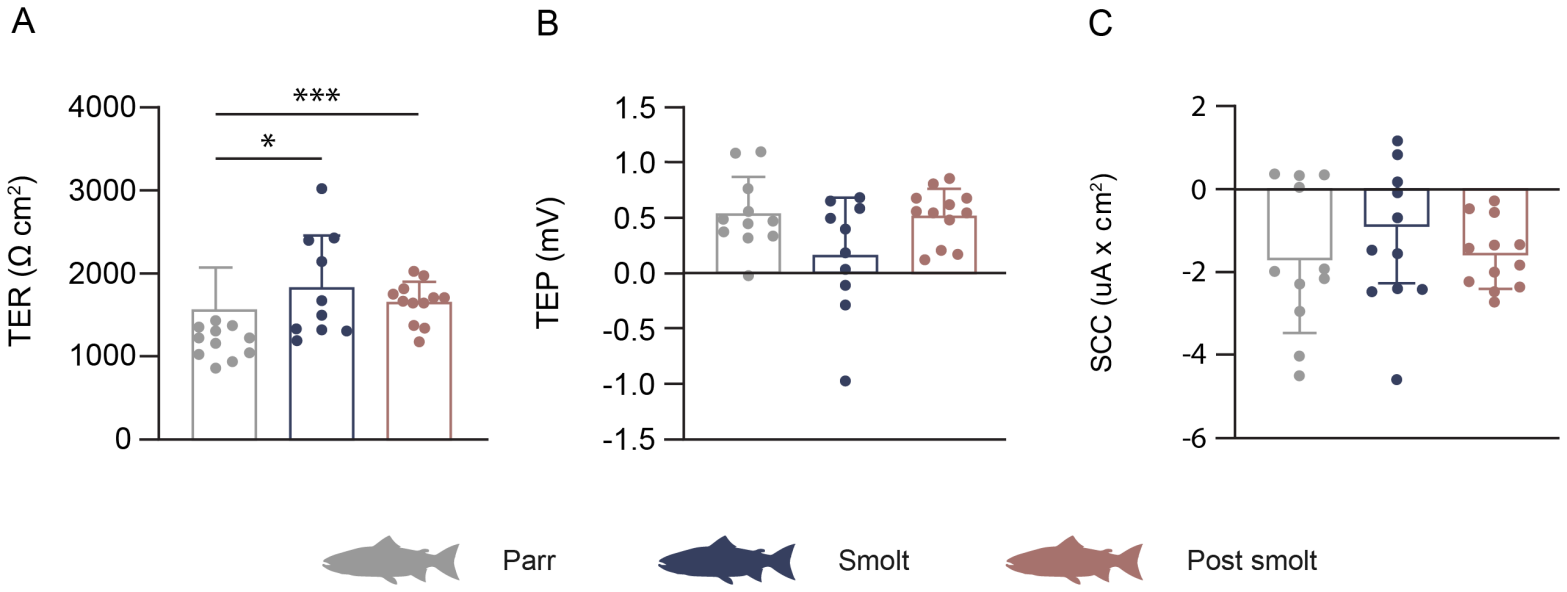
A comparison of skin **(A)** transepithelial electrical resistance (TER), **(B)** transepithelial potential difference (TEP), and **(C)** short-circuit current (SCC) between three different Atlantic salmon life stages, parr, smolt and post-smolt, under identical Ringer`s conditions (parr n = 11, smolt n = 10, post-smolt n = 12). Bars represent mean ± SD. *P ≤ 0.05, ***P ≤ 0.001.

Welch’s ANOVA showed significant differences in skin TER between fish at different life stages under identical Ringer`s conditions (W_2, 17.26_ = 15.71, *P* = 0.0073; **Figure 1A**). Dunnett’s T3 multiple comparison tests showed that parr had significantly lower skin TER than both smolt (*P* = 0.0280) and post-smolt (*P* < 0.0001). There were no significant differences in TEP or SCC between any group (**Figures 1B, C**). In all cases, the TEP was positive in relation to the basal side, indicating more positive ions on the apical side.

### Electrophysiology under *in vivo* like conditions

**Figure 2:**
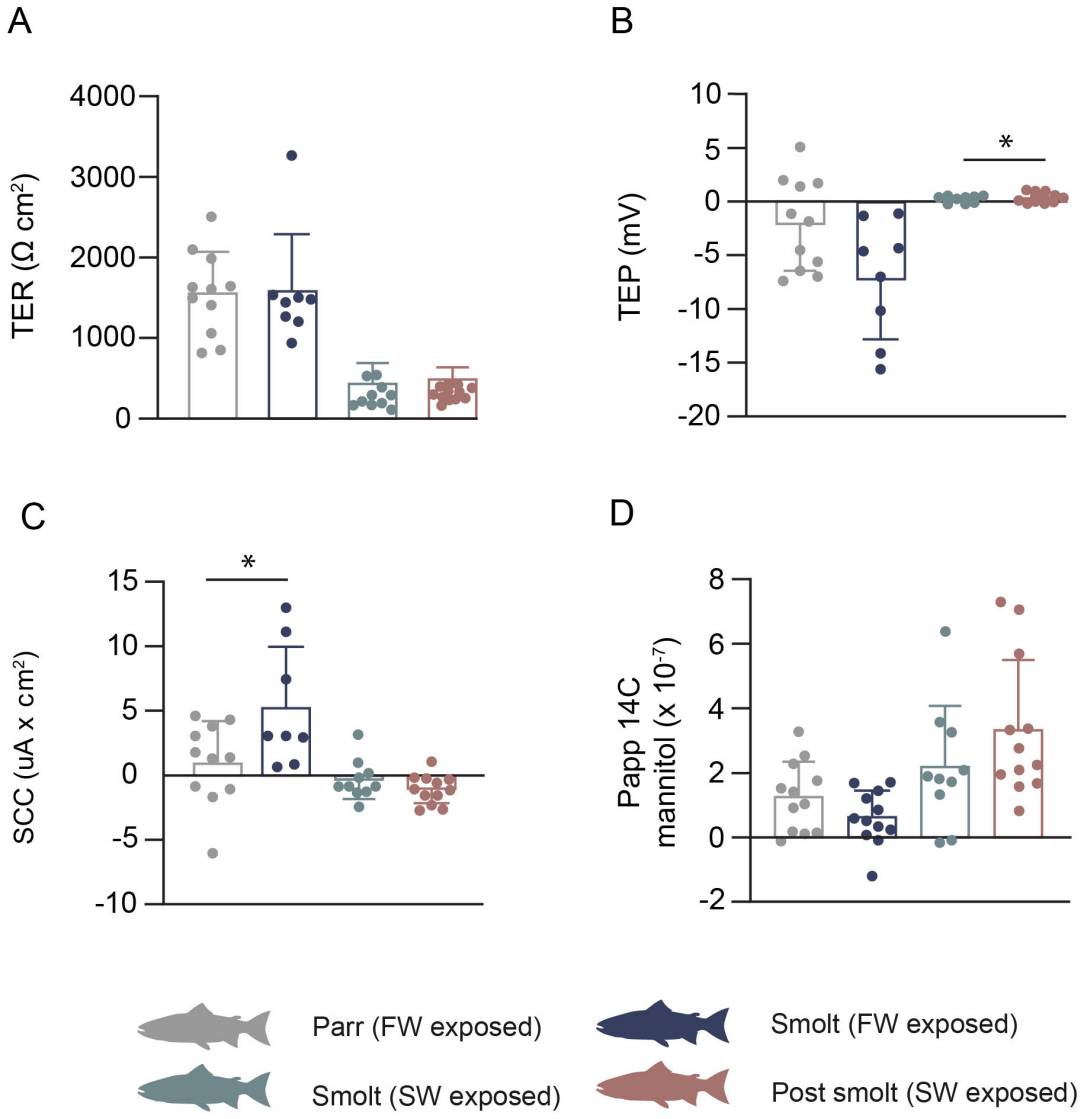
A comparison of skin **(A)** transepithelial electrical resistance (TER), **(B)** transepithelial potential difference (TEP), **(C)** short-circuit current (SCC) and **(D)** Papp 14C mannitol between three different Atlantic salmon life stages, parr, smolt and post-smolt, following exposure to either FW or SW on the apical side (n = 8-12). Bars represent mean and SD. *P ≤0.05.

Exposure to FW in parr and smolt resulted in a drastic increase in TER compared to identical Ringer´s conditions, which corresponds to a sharp reduction in epithelial permeability. Following exposure to FW, the mean TER of the parr and smolt both exceeded 1500 Ω cm^2^. There were no significant differences in TER (**Figure 2A**) or TEP (**Figure 2B**) between parr and smolt following exposure to FW. However, Welch’s test showed that smolt exposed to FW had significantly higher SCC (in absolute values; **Figure 2C**) compared to parr exposed to FW (t_11.50_ = 2.239, *P* = 0.0458).

Exposure to SW in smolt and post-smolt resulted in a reduction in TER compared to identical Ringer´s conditions, which corresponds to an increase in epithelial permeability. There were no significant differences in TER (**Figure 2A**) or SCC (**Figure 2C**) between smolt and post-smolt following exposure to SW. However, the post-smolt exposed to SW had significantly higher TEP than the smolt exposed to SW (t_18.86_ = 2.192, *P* = 0.0411; **Figure 2B**). For the SW exposed fish, both TEP and SCC displayed the opposite polarity compared to the FW exposed fish. Permeability to 14C mannitol did not differ significantly between groups (**Figure 2D**).

### Gene expression analysis

**Figure 3:**
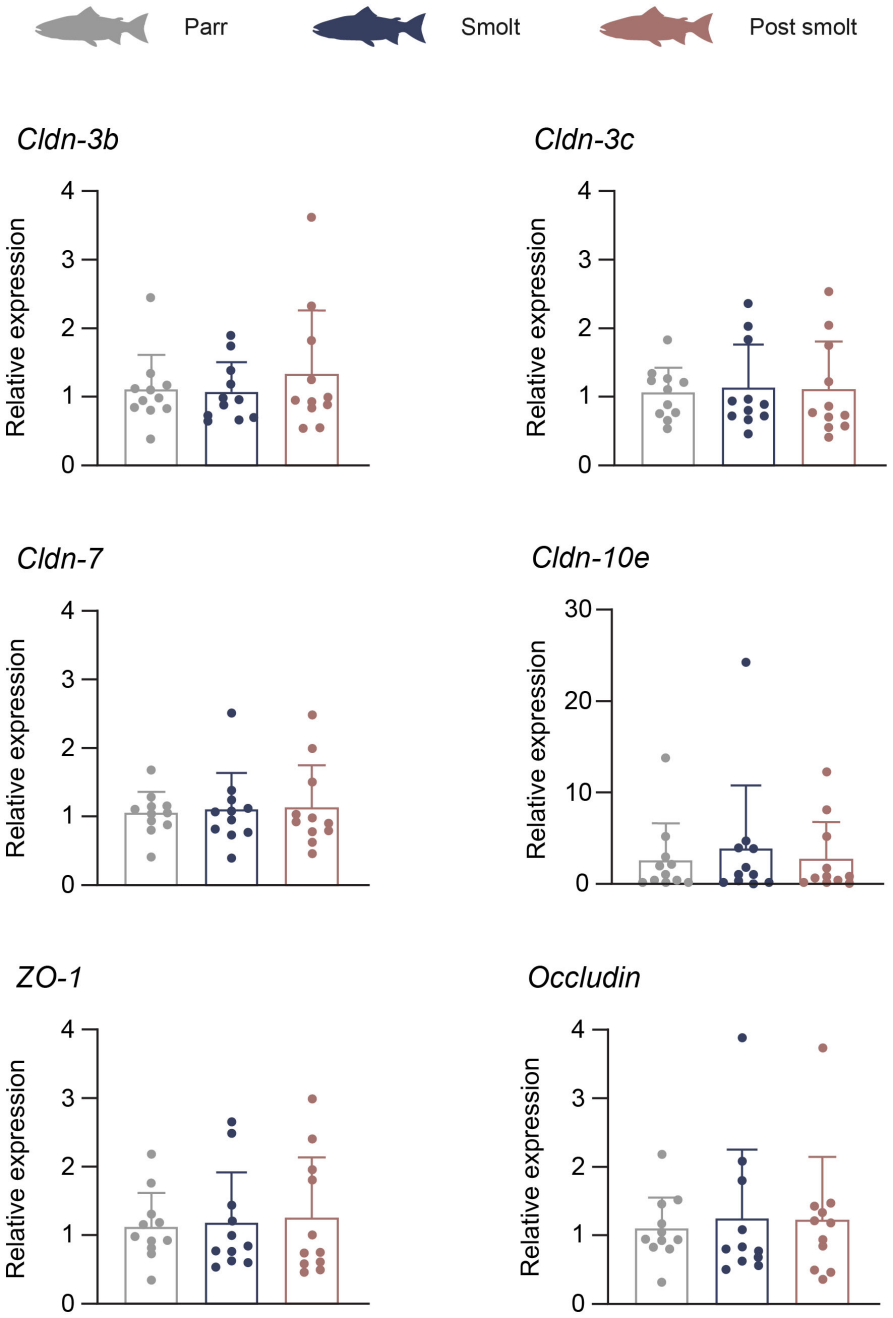
Relative mRNA expression of tight junction proteins throughout smoltification (n = 11). The data were normalized using *ef-1a* as a reference gene, based on Tipsmark et al. (2008). Data are expressed as mean and SD. No significant differences were detected based on Welch ANOVAs.

Of the four claudins that were included in the study, all were found to be present in the skin of Atlantic salmon. When comparing the three different life stages, no significant differences in expression of any of the genes were found (**Figure 3**). Additionally, no significant correlation was detected between TER and mRNA expression of any of the genes used in the current study (**Supplementary Figures S1**, **S2**).

## Discussion

The skin acts as a critical barrier that separates the internal environment of the fish from its external environment. As such, skin permeability plays an essential role in preventing passive water and ion fluxes, as well as limiting pathogen entry. In rainbow trout, skin permeability varies depending on environmental salinity, with exposure to SW generally increasing the epithelial leakiness (Doyle et al., 2022). However, the dynamic regulation of skin barrier function throughout smoltification, and how permeability responds to acute changes in environmental salinity at different life stages, has remained poorly characterized. The present study provides new insights by demonstrating that skin permeability in Atlantic salmon changes both across developmental stages and in response to environmental salinity, highlighting the flexible and responsive nature of the skin barrier during smoltification.

### Skin function at identical Ringer's conditions

Investigation of skin permeability at identical Ringer´s conditions revealed significantly lower TER in the skin of the parr compared to that of both smolt and post-smolt. This corresponds to a tighter skin barrier in smolt and post-smolt. The exact reason for these differences in ionic permeability is unclear. However, the differences observed in skin TER throughout smoltification are somewhat similar to those observed in the intestine, where intestinal TER is higher in post-smolt in SW than in the parr in FW when assessed at identical Ringer’s conditions (Sundell et al., 2003). In the intestine, cortisol has been shown to be the main endocrine regulator of intestinal fluid transport increase during smoltification (Sundell and Sundh, 2012; Veillette et al., 1995). As cortisol has previously been shown to alter the expression of skin claudins in rainbow trout (Gauberg et al., 2017), it is possible that cortisol may play a similar role in the skin, though this was not directly measured in the current study and remains speculative. The changes in barrier function observed at identical Ringer’s conditions suggest some change in the skin components that govern epithelial permeability, most likely involving the tight junctions and claudins, though the precise mechanisms remain to be determined.

At identical Ringer´s conditions, both TEP and SCC were low across all life stages, supporting the idea that the skin, while capable of active transport, primarily functions as a diffusion barrier. This observation aligns with previous findings in rainbow trout skin (Doyle et al., 2022). The absence of significant TEP differences between parr, smolt, and post-smolt suggests that the overall electrochemical environment across the epithelium is preserved despite differences in epithelial tightness and morphology at different life stages. The significant reduction in SCC at the smolt stage compared to parr may be linked to this increased barrier tightness and may relate to developmental changes in ion selectivity and/or tight junction composition.

### Skin function under *in-vivo*-like conditions

The dramatic increase observed in TER after addition of FW to the apical compartment to simulate *in vivo* conditions was similar in parr and FW smolts. On the contrary, SW exposure appeared to reduce barrier function as evident by the reduction in TER of FW smolts and SW post-smolts. This confirms the strong regulating role of environmental salinity on skin permeability, which was previously observed in rainbow trout (Doyle et al., 2022). The observed increase in TER under FW exposure aligns with the general epithelial response pattern reported in euryhaline organisms, where tight epithelial barriers are needed in FW to minimize passive ion loss and fluid gain (Kirschner, 1991). Further, this highlights that the salmon skin is similar to the epithelia of euryhaline organisms moving from FW to SW (reviewed by Henry et al., 2012; Sundell et al., 2019).

As mentioned, permeability to ions is controlled and regulated by the tight junctions, particularly the claudins. Most knowledge on the function of TJs in fish and their response to environmental salinity comes from studies of the gill. Permeability in the gill and the opercular skin is partly believed to be determined by the depth of the tight junctions. In FW, “deep” tight junctions (300–500 nm) are characterized as relatively impermeable linking the mitochondria rich cells (MRCs) to adjacent pavement cells (PVCs; Karnaky, 1986). This contrasts with the higher leakiness in the SW gill, which coincides with shallow (~15 nm) TJs that link MRCs to neighboring accessory cells (ACs) through which the Na^+^ is shunted during osmoregulatory ion secretion (Marshall, 2002; Marshall and Bryson, 1998; Karnaky, 1986; Sardet et al., 1979). The skin of adult fish is ostensibly devoid of accessory cells and mitochondria rich cells (with the exception of opercular/cleithrum skin and the general integument of some marine and amphibious species; Karnaky and Kinter, 1977; Marshall et al., 1992). However, the structure of TJs in the skin, and whether they can be characterized as shallow or deep, remains unclear for now.

Alterations in epithelial permeability in response to salinity have previously been shown to occur rapidly, in the gill (Chasiotis et al., 2012) and skin (Doyle et al., 2022). Again, this is likely due to acute changes in the structure and function of the TJs. The precise mechanisms behind acute TJ remodeling in teleost fish remain unclear, and were not directly investigated in the current study. Evidence from other systems may offer some insight. In the goldfish intestine, a rapid cAMP-regulated increase in TJ conductance for chloride has been described (Bakker and Groot, 1989), while studies on mammalian cells suggest that TJs are dynamic structures capable of rapid remodeling through phosphorylation and/or endocytosis of TJ proteins (Shen et al., 2008; Stamatovic et al., 2017; Van Itallie and Anderson, 2006), with responses occurring within minutes (González-Mariscal et al., 2008). Whether analogous mechanisms operate in Atlantic salmon skin is currently unknown. Nevertheless, such mechanisms would be consistent with the rapid changes in TER observed in the current study following acute salinity shifts, as well as those reported in trout and tilapia gill cell lines (Wood et al., 2002), and represent a plausible avenue for future investigation.

The drastic changes in TER following the alteration of environmental salinity were accompanied by changes in TEP and SCC. Although active transport in salmon skin is generally low, as seen at identical Ringer`s conditions, there is a consistent basal-positive potential. The reversal to a more negative TEP upon FW exposure is therefore likely driven by increased passive, basal-to-apical diffusion of cations (presumably Na^+^). This passive cation efflux also likely explains the concurrently higher SCC. In summary, the observed changes in TEP and SCC in FW appear to be primarily a consequence of altered passive paracellular permeability, rather than a change in active transcellular transport. It is also important to note the temperature differences at the different life stages, which may have influenced membrane fluidity and ion transport. However, as chamber temperature was maintained at the respective tank temperatures, the conditions reflect the actual physiological state of the fish at the time of sampling.

### Changes in the mRNA expression of skin TJ proteins during smoltification

As the claudins are the primary components of ion selectivity in the TJs, we expected to observe significant differences in the claudin repertoire throughout smoltification, which would reflect the pre-adaptive and adaptive needs in parr, smolt and post-smolt, respectively. Contrary to expectation, there were no significant differences in the expression of any of the examined TJ proteins between Atlantic salmon life stages. This may suggest a general requirement for the selected claudins at each life stage. The lack of statistically significant differences in claudin mRNA expression between different life stages makes it impossible to conclusively comment on the role of the claudins analyzed in this study.

However, there was a tendency toward differential mRNA expression of several genes at different life stages, which seems consistent with previous work in other epithelia. For example, Tipsmark et al. (2008) observed a peak in branchial expression of cldn-10e, corresponding to the period of peak smoltification. In the intestine, Tipsmark et al. (2010) showed changes in the mRNA expression of *cldns* -*15* and -*25b*. In the present study, cldn-10e expression was highest in skin from smolts, suggesting similarities in expression between the skin and gill for this particular isoform in that life stage. However, branchial expression of cldn-10e has been shown to elevate following SW transfer in several species (Bossus et al., 2015; Bui and Kelly, 2014) including Atlantic salmon (Tipsmark et al., 2008), and this was not observed in the skin in the current study. Claudin 10e (and the claudin 10 family as a whole) is thought to be involved in the creation and maintenance of cation selective pores, suggesting a conserved role in salt secreting epithelia (Marshall et al., 2018). In relation to the isoforms from the claudin 3 family (cldns-3a, -3b and -3c), there appeared again to be some similarities in expression between gill and skin in Atlantic salmon. Previously, cldn-3a was shown to be expressed abundantly in the kidney and liver, and to a lesser extent in the intestine (Tipsmark and Madsen, 2012). However, as with the skin in the current study, cldn-3a was not detected in the gill. The low expression of cldn-3b in the current study is consistent with that observed in the gill. However, cldn-3c is abundantly expressed in the gill, while low expression was observed in the skin. Though not significant, both cldn-3 isoforms showed a tendency to higher expression in the SW acclimated fish. The cldn-3 family is thought to be involved in renal remodeling in SW environments and so a similar process may occur in the skin. The exact role of the claudin-3 family in fish is unclear. However, evidence from MDCK cells transfected with human claudin-3 suggests a role in tightening the paracellular pathway, and thus increasing transepithelial resistance (Milatz et al., 2010).

There was no correlation between TER and the relative mRNA expression of any of the barrier function related genes examined in this study. This was interesting because the skin exhibits a large degree of within-group variation in TER, when compared to e.g. the intestine (Sundell and Sundh, 2012). Previously, we hypothesized (Doyle et al., 2022) that the differential expression of claudin isoforms might contribute to this variation. However, we could not confirm this in the present study for Atlantic salmon. It is important to highlight that we examined a limited set of claudin isoforms selected based on studies of Atlantic salmon gill (Tipsmark et al., 2009; Tipsmark and Madsen, 2012), and further research including a broader range of claudins may be required. Despite many similarities in expression pattern, as the skin fulfills many functions that differ from those of gill, it likely also expresses a different suite of claudins/TJ proteins. In the case of rainbow trout, claudin expression in the skin differs markedly based on skin region and in response to dietary cortisol (Gauberg et al., 2017). This suggests that the skin has a need for a differential expression of claudins and that this expression is mediated, at least in part, by endocrine factors. Thus, an avenue for future research regarding Atlantic salmon skin TJs may be to explore analogous isoforms that are shown to be highly expressed in rainbow trout skin e.g. cldns -30, 32a, and 38b. This may shed more light on the possible involvement of different TJ components in the differential barrier function during smoltification observed in the current study. It is also important to highlight that while claudins play a critical role in the function of the TJs, they also play a role in general cell-cell interactions (Hagen, 2017). Thus, different claudin isoforms may occur between cells throughout the epithelium and, rather than co-localize with occludin and ZO-1 to form a functional barrier, they may ‘float’ within the cell membrane. As such, the skin may have several claudin isoforms that are constitutively expressed, while other, specific isoforms, may be rapidly inserted into the TJs in response to varying environmental conditions.

## Conclusion

This study demonstrates that Atlantic salmon skin undergoes both developmental and acute functional modifications during smoltification and seawater acclimation. The increase in TER from parr to post-smolt under identical Ringer`s conditions indicates a tighter skin barrier. Further, the rapid changes in TER, TEP, and SCC following acute salinity shifts highlight TJs as highly dynamic structures capable of immediate remodeling. Despite these clear functional adjustments, the mRNA expression of the selected claudins and TJ-associated proteins did not differ significantly across life stages, suggesting that additional claudin isoforms, other TJ components, or post-transcriptional mechanisms (or a combination of these) are responsible for the observed modulation of skin barrier function. Taken together, these findings suggest that the skin in Atlantic salmon is primarily a protective barrier with limited active transport, yet capable of rapid physiological adjustments.

## Data Availability

The data presented in the study are deposited in the Figshare repository, DOI: https://doi.org/10.6084/m9.figshare.32680821.

## References

[B1] Ángeles Esteban M. (2012). An overview of the immunological defenses in fish skin. Int. Scholarly. Res. Notices. 2012, 853470. doi: 10.5402/2012/853470 42258293

[B2] Bakker R. Groot J. A. (1989). Further evidence for the regulation of the tight junction ion selectivity by cAMP in goldfish intestinal mucosa. J. Membr. Biol. 111, 25–35. doi: 10.1007/bf01869206 30311153

[B3] Björnsson B. T. Stefansson S. O. McCormick S. D. (2011). Environmental endocrinology of salmon smoltification. Gen. Comp. Endocrinol. 170, 290–298. doi: 10.1016/j.ygcen.2010.07.003 20627104

[B4] Bossus M. C. Madsen S. S. Tipsmark C. K. (2015). Functional dynamics of claudin expression in Japanese medaka (Oryzias latipes): Response to environmental salinity. Comp. Biochem. Physiol. Part. A. Mol. Integr. Physiol. 187, 74–85. doi: 10.1016/j.cbpa.2015.04.017 25957710

[B5] Bui P. Kelly S. P. (2014). Claudin-6, -10d and -10e contribute to seawater acclimation in the euryhaline puffer fish Tetraodon nigroviridis. J. Exp. Biol. 217, 1758–1767. doi: 10.1242/jeb.099200 24526724

[B6] Chasiotis H. Kolosov D. Bui P. Kelly S. P. (2012). Tight junctions, tight junction proteins and paracellular permeability across the gill epithelium of fishes: A review. Respir. Physiol. Neurobiol. 184, 269–281. doi: 10.1016/j.resp.2012.05.020 22640933

[B7] Cummins P. M. (2012). Occludin: One protein, many forms. Mol. Cell. Biol. 32, 242–250. doi: 10.1128/mcb.06029-11 22083955 PMC3255790

[B8] Doyle D. Carney Almroth B. Sundell K. Simopoulou N. Sundh H. (2022). Transport and barrier functions in rainbow trout trunk skin are regulated by environmental salinity. Front. Physiol. 13. doi: 10.3389/fphys.2022.882973 35634157 PMC9136037

[B9] Fagan M. S. O’Byrne-Ring N. Ryan R. Cotter D. Whelan K. Mac Evilly U. (2003). A biochemical study of mucus lysozyme, proteins and plasma thyroxine of Atlantic salmon (Salmo salar) during smoltification. Aquaculture 222, 287–300. doi: 10.1016/s0044-8486(03)00128-5

[B10] Gauberg J. Kolosov D. Kelly S. P. (2017). Claudin tight junction proteins in rainbow trout (Oncorhynchus mykiss) skin: Spatial response to elevated cortisol levels. Gen. Comp. Endocrinol. 240, 214–226. doi: 10.1016/j.ygcen.2016.10.006 27771288

[B11] Glover C. N. Bucking C. Wood C. M. (2013). The skin of fish as a transport epithelium: A review. J. Comp. Physiol. B. 183, 877–891. doi: 10.1007/s00360-013-0761-4 23660826

[B12] Gomes A. S. Balseiro P. Iversen M. D. Zimmermann F. Shimizu M. Izutsu A. . (2025). Performance of Atlantic salmon reared under three different regimes of continuous aerobic exercise during the freshwater phase. Aquaculture 609, 742797. doi: 10.1016/j.aquaculture.2025.742797 38826717

[B13] González-Mariscal L. Tapia R. Chamorro D. (2008). Crosstalk of tight junction components with signaling pathways. Biochim. Biophys. Acta 1778, 729–756. doi: 10.1016/j.bbamem.2007.08.018 17950242

[B14] Grosell M. (2006). Intestinal anion exchange in marine fish osmoregulation. J. Exp. Biol. 209, 2813–2827. doi: 10.1242/jeb.02345 16857865

[B15] Guttman J. A. Finlay B. B. (2009). Tight junctions as targets of infectious agents. Biochim. Biophys. Acta 1788, 832–841. doi: 10.1016/j.bbamem.2008.10.028 19059200

[B16] Hagen S. J. (2017). Non-canonical functions of claudin proteins: beyond the regulation of cell-cell adhesions. Tissue Barriers. 5, e1327839. doi: 10.1080/21688370.2017.1327839 28548895 PMC5501131

[B17] Hartsock A. Nelson W. J. (2008). Adherens and tight junctions: structure, function and connections to the actin cytoskeleton. Biochim. Biophys. Acta (BBA)-Biomembr. 1778, 660–669. doi: 10.1016/j.bbamem.2007.07.012 17854762 PMC2682436

[B18] Henry R. P. Lucu C. Onken H. Weihrauch D. (2012). Multiple functions of the crustacean gill: Osmotic/ionic regulation, acid-base balance, ammonia excretion, and bioaccumulation of toxic metals. Front. Physiol. 3, 1–33. doi: 10.3389/fphys.2012.00431 23162474 PMC3498741

[B19] Karlsen C. Ytteborg E. Timmerhaus G. Høst V. Handeland S. Jørgensen S. M. . (2018). Atlantic salmon skin barrier functions gradually enhance after seawater transfer. Sci. Rep. 8, 9510. doi: 10.1038/s41598-018-27818-y 29934588 PMC6015023

[B20] Karnaky K. J. (1986). Structure and function of the chloride cell of Fundulus heteroclitus and other teleosts. Am. Zool. 26, 209–224. doi: 10.1093/icb/26.1.209

[B21] Karnaky K. J. Kinter W. B. (1977). Killifish opercular skin: A flat epithelium with a high density of chloride cells. J. Exp. Zool. 199, 355–364. doi: 10.1002/jez.1401990309 850116

[B22] Kirschner L. B. (1991). Water and ions. In: Prosser L. (Ed.), Environmental and Metabolic Animal Physiology. (London: Wiley-Liss) pp. 13–107.

[B23] Kolosov D. Bui P. Chasiotis H. Kelly S. P. (2013). Claudins in teleost fishes. Tissue Barriers. 1, e25391. doi: 10.4161/tisb.25391 24665402 PMC3875606

[B24] Krause G. Winkler L. Mueller S. L. Haseloff R. F. Piontek J. Blasig I. E. (2008). Structure and function of claudins. Biochim. Biophys. Acta 1778, 631–645. doi: 10.1016/j.bbamem.2007.10.018 18036336

[B25] Krogh A. (1938). The active absorption of ions in some freshwater animals. Z. Fr. vergleichende. Physiol. 25, 335–350. doi: 10.1007/bf00339641 30311153

[B26] Loh Y. H. Christoffels A. Brenner S. Hunziker W. Venkatesh B. (2004). Extensive expansion of the claudin gene family in the teleost fish, Fugu rubripes. Genome Res. 14, 1248–1255. doi: 10.1101/gr.2400004 15197168 PMC442139

[B27] Marshall W. S. (2002). Na+, Cl-, Ca2+ and Zn2+ transport by fish gills: Retrospective review and prospective synthesis. J. Exp. Zool. 293, 264–283. doi: 10.1002/jez.10127 12115901

[B28] Marshall W. S. Breves J. P. Doohan E. M. Tipsmark C. K. Kelly S. P. Robertson G. N. . (2018). Claudin-10 isoform expression and cation selectivity change with salinity in salt-secreting epithelia of Fundulus heteroclitus. J. Exp. Biol. 221, jeb168906. doi: 10.1242/jeb.168906 29150449

[B29] Marshall W. S. Bryson S. E. (1998). Transport mechanisms of seawater teleost chloride cells: An inclusive model of a multifunctional cell. Comp. Biochem. Physiol. Part. A. Mol. Integr. Physiol. 119, 97–106. doi: 10.1016/s1095-6433(97)00402-9 11253824

[B30] Marshall W. S. Bryson S. E. Wood C. M. (1992). Calcium transport by isolated skin of rainbow trout. J. Exp. Biol. 166, 297–316. doi: 10.1242/jeb.166.1.297 1602278

[B31] Milatz S. Krug S. M. Rosenthal R. Günzel D. Müller D. Schulzke J.-D. . (2010). Claudin-3 acts as a sealing component of the tight junction for ions of either charge and uncharged solutes. Biochim. Biophys. Acta 1798, 2048–2057. doi: 10.1016/j.bbamem.2010.07.014 20655293

[B32] Pfaffl M. W. (2001). A new mathematical model for relative quantification in real-time RT–PCR. Nucleic Acids Res. 29, e45. doi: 10.1093/nar/29.9.e45 11328886 PMC55695

[B33] Rakers S. Gebert M. Uppalapati S. Meyer W. Maderson P. Sell A. F. . (2010). Fish matters”: The relevance of fish skin biology to investigative dermatology. Exp. Dermatol. 19, 313–324. doi: 10.1111/j.1600-0625.2009.01059.x 20158518

[B34] Sardet C. Pisam M. Maetz J. (1979). The surface epithelium of teleostean fish gills. Cellular and junctional adaptations of the chloride cell in relation to salt adaptation. J. Cell Biol. 80, 96–117. doi: 10.1083/jcb.80.1.96 422655 PMC2110284

[B35] Shen L. Weber C. R. Turner J. R. (2008). The tight junction protein complex undergoes rapid and continuous molecular remodeling at steady state. J. Cell Biol. 181, 683–695. doi: 10.1083/jcb.200711165 18474622 PMC2386107

[B36] Stamatovic S. M. Johnson A. M. Sladojevic N. Keep R. F. Andjelkovic A. V. (2017). Endocytosis of tight junction proteins and the regulation of degradation and recycling. Ann. N. Y. Acad. Sci. 1397, 54–65. doi: 10.1111/nyas.13346 28415156 PMC5479724

[B37] Sundell K. Jutfelt F. Ágústsson T. Olsen R.-E. Sandblom E. Hansen T. . (2003). Intestinal transport mechanisms and plasma cortisol levels during normal and out-of-season parr–smolt transformation of Atlantic salmon, Salmo salar. Aquaculture 222, 265–286. doi: 10.1016/s0044-8486(03)00127-3

[B38] Sundell K. S. Sundh H. (2012). Intestinal fluid absorption in anadromous salmonids: Importance of tight junctions and aquaporins. Front. Physiol. 3, 388. doi: 10.3389/fphys.2012.00388 23060812 PMC3460234

[B39] Sundell K. Wrange A. L. Jonsson P. R. Blomberg A. (2019). Osmoregulation in barnacles: an evolutionary perspective of potential mechanisms and future research directions. Front. Physiol. 10, 877. doi: 10.3389/fphys.2019.00877 31496949 PMC6712927

[B40] Sveen L. R. Robinson N. Krasnov A. Daniels R. R. Vaadal M. Karlsen C. . (2023). Transcriptomic landscape of Atlantic salmon (Salmo salar L.) skin. G3.: Genes|Genomes|Genet. 13, jkad215. doi: 10.1093/g3journal/jkad215 37724757 PMC10627282

[B41] Sveen L. R. Timmerhaus G. Torgersen J. S. Ytteborg E. Jørgensen S. M. Handeland S. . (2016). Impact of fish density and specific water flow on skin properties in Atlantic salmon (Salmo salar L.) post-smolts. Aquaculture 464, 629–637. doi: 10.1016/j.aquaculture.2016.08.012 38826717

[B42] Takano K. Kojima T. Sawada N. Himi T. (2014). Role of tight junctions in signal transduction: An update. EXCLI. J. 13, 1145–1162. 26417329 PMC4464418

[B43] Tipsmark C. K. Breves J. P. Rabeneck D. B. Trubitt R. T. Lerner D. T. Grau E. G. (2016). Regulation of gill claudin paralogs by salinity, cortisol and prolactin in Mozambique tilapia (Oreochromis mossambicus). Comp. Biochem. Physiol. Part. A. Mol. Integr. Physiol. 199, 78–86. doi: 10.1016/j.cbpa.2016.05.014 27210417

[B44] Tipsmark C. K. Jørgensen C. Brande-Lavridsen N. Engelund M. Olesen J. H. Madsen S. S. (2009). Effects of cortisol, growth hormone and prolactin on gill claudin expression in Atlantic salmon. Gen. Comp. Endocrinol. 163, 270–277. doi: 10.1016/j.ygcen.2009.04.020 19401202

[B45] Tipsmark C. K. Kiilerich P. Nilsen T. O. Ebbesson L. O. E. Stefansson S. O. Madsen S. S. (2008). Branchial expression patterns of claudin isoforms in Atlantic salmon during seawater acclimation and smoltification. Am. J. Physiol. Regul. Intgr. Comp. Physiol. 294, R1563–R1574. doi: 10.1152/ajpregu.00915.2007 18321951

[B46] Tipsmark C. K. Madsen S. S. (2012). Tricellulin, occludin and claudin-3 expression in salmon intestine and kidney during salinity adaptation. Comp. Biochem. Physiol. Part. A. Mol. Integr. Physiol. 162, 378–385. doi: 10.1016/j.cbpa.2012.04.020 22561661

[B47] Tipsmark C. K. Sørensen K. J. Hulgard K. Madsen S. S. (2010). Claudin-15 and -25b expression in the intestinal tract of Atlantic salmon in response to seawater acclimation, smoltification and hormone treatment. Comp. Biochem. Physiol. Part. A. Mol. Integr. Physiol. 155, 361–370. doi: 10.1016/j.cbpa.2009.11.025 19969100

[B48] Van Itallie C. M. Anderson J. M. (2006). Claudins and epithelial paracellular transport. Annu. Rev. Physiol. 68, 403–429. doi: 10.1146/annurev.physiol.68.040104.131404 16460278

[B49] Veillette P. A. Sundell K. Specker J. L. (1995). Cortisol mediates the increase in intestinal fluid absorption in Atlantic salmon during parr-smolt transformation. Gen. Comp. Endocrinol. 97, 250–258. doi: 10.1006/gcen.1995.1024 7622019

[B50] Wood C. M. Kelly S. P. Zhou B. Fletcher M. O’Donnell M. Eletti B. . (2002). Cultured gill epithelia as models for the freshwater fish gill. Biochim. Biophys. Acta (BBA). - Biomembr. 1566, 72–83. doi: 10.1016/s0005-2736(02)00595-3 12421539

[B51] Zihni C. Mills C. Matter K. Balda M. S. (2016). Tight junctions: From simple barriers to multifunctional molecular gates. Nat. Rev. Mol. Cell Biol. 17, 564–580. doi: 10.1038/nrm.2016.80 27353478

[B52] Zimmer A. M. Brauner C. J. Wood C. M. (2014). Ammonia transport across the skin of adult rainbow trout (Oncorhynchus mykiss) exposed to high environmental ammonia (HEA). J. Comp. Physiol. B. 184, 77–90. doi: 10.1007/s00360-013-0784-x 24114656

