# Supplementary material for: Variation in skin barrier function throughout smoltification in Atlantic salmon (Salmo salar)
Source: Front Physiol. 2026 Jun 19;17:1856527. doi: 10.3389/fphys.2026.1856527 (PMC13328031; doi:10.3389/fphys.2026.1856527)
Supplement: Supplementary file 1 [file SupplementaryFile1.docx]

**Supplementary material for ‘Variation in skin barrier function throughout smoltification in Atlantic salmon (*Salmo salar*)’**


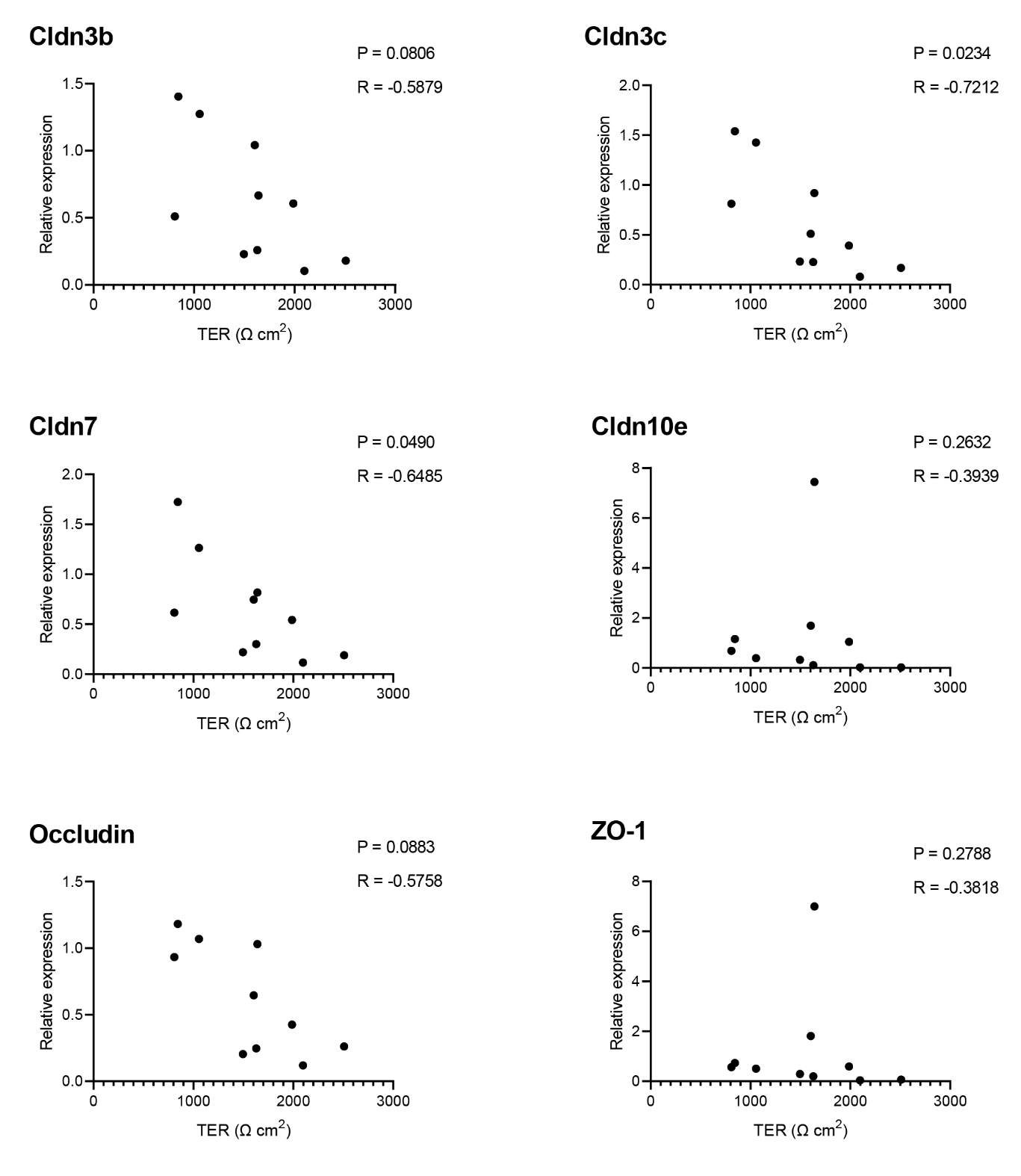


Figure S1. Spearman rank correlation between mRNA expression of selected tight junction genes and transepithelial electrical resistance (TER) in Atlantic salmon skin exposed to freshwater. Correlations are shown for *Cldn-3b*, *Cldn-3c*, *Cldn-7*, *Cldn-10e*, *Occludin*, and *ZO-1* (n = 10). The significance threshold was adjusted for multiple comparisons using Bonferroni correction (adjusted α = 0.0083).


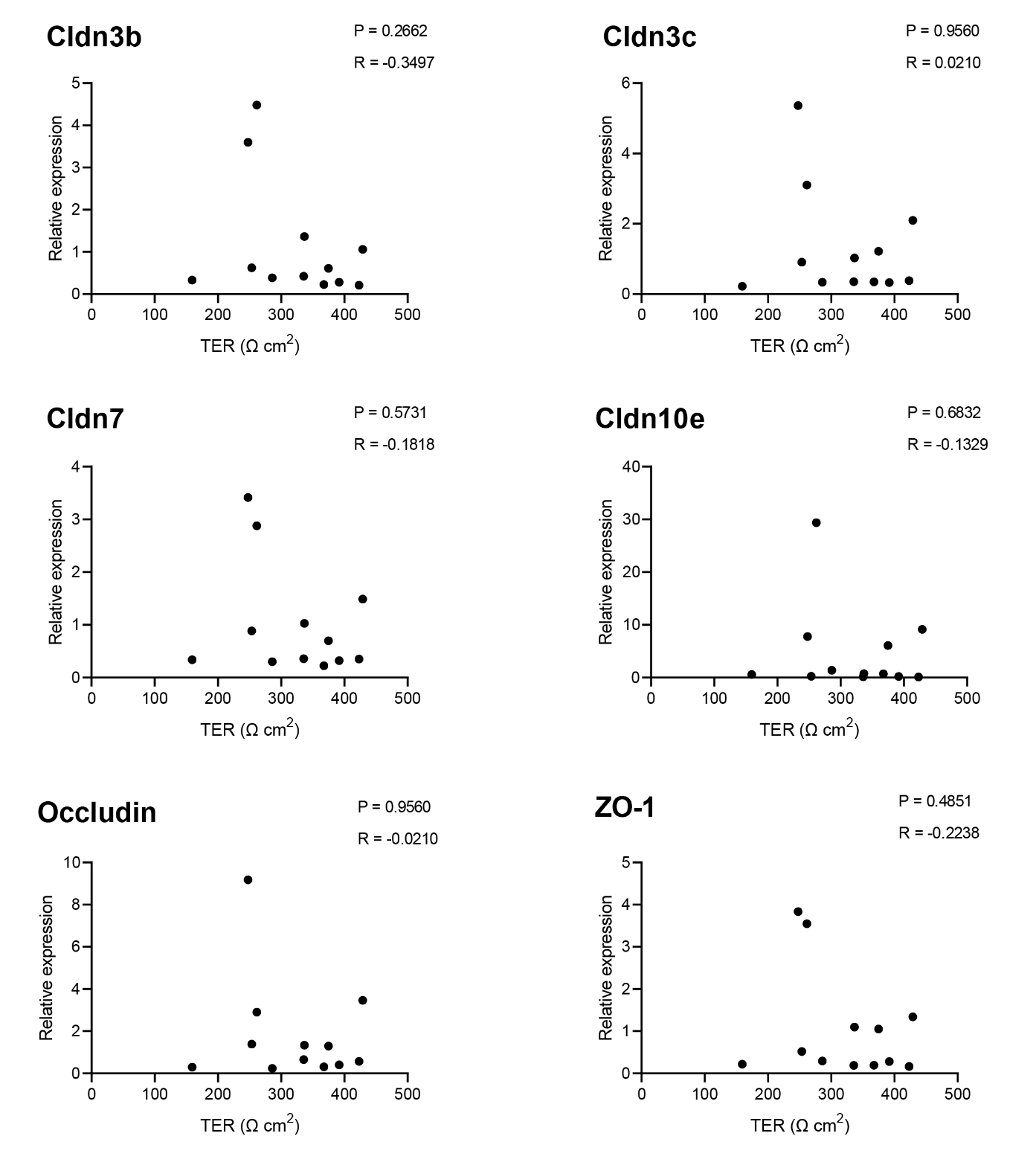


Figure S2. Spearman rank correlation between mRNA expression of selected tight junction genes and transepithelial electrical resistance (TER) in Atlantic salmon skin exposed to seawater. Correlations are shown for *Cldn-3b*, *Cldn-3c*, *Cldn-7*, *Cldn-10e*, *Occludin*, and *ZO-1* (n = 12). The significance threshold was adjusted for multiple comparisons using Bonferroni correction (adjusted α = 0.0083).
